# *Vipr2* Gene Expression Is Upregulated in the Nucleus Accumbens of Spontaneous Hypertensive Rats During Early Life

**DOI:** 10.1002/npr2.70143

**Published:** 2026-06-17

**Authors:** Ryota Araki, Ayami Kita, Yukio Ago, Takeshi Yabe

**Affiliations:** ^1^ Laboratory of Functional Biomolecules and Chemical Pharmacology, Faculty of Pharmaceutical Sciences Setsunan University Hirakata Osaka Japan; ^2^ Department of Cellular and Molecular Pharmacology, Graduate School of Biomedical and Health Sciences Hiroshima University Hiroshima Japan; ^3^ Global Center for Medical Engineering and Informatics The University of Osaka Suita Osaka Japan

**Keywords:** ADHD, DNA methylation, nucleus accumbens, spontaneous hypertensive rat, VIPR2

## Abstract

**Aim:**

Attention‐deficit/hyperactivity disorder (ADHD) is one of the most common neurodevelopmental disorders. Recent methylome analyses have shown that peripheral DNA methylation levels in the *VIPR2* gene, which encodes vasoactive intestinal peptide receptor 2, are correlated with ADHD symptoms. However, mRNA expression and DNA methylation levels of the *VIPR2* gene in the brain in ADHD are unknown. In this study, we examined the mRNA expression and DNA methylation levels of the *Vipr2* gene in the brains of spontaneous hypertensive rats (SHRs), which are used as an animal model of ADHD.

**Methods:**

The mRNA levels of *Vipr2* and related genes were analyzed in the prefrontal cortex, nucleus accumbens (NAc), amygdala, and hippocampus of SHRs and Wistar Kyoto rats (WKYs), a reference strain, by quantitative reverse transcription polymerase chain reaction. DNA methylation levels at the cytosine–phosphate–guanine (CpG) islands of the *Vipr2* gene were evaluated in the NAc of WKYs and SHRs by bisulfite sequencing.

**Results:**

The mRNA level of *Vipr2* was significantly higher in the NAc, but not in the prefrontal cortex, amygdala, or hippocampus, of male SHRs compared with male WKYs at 4 weeks old. Upregulation of the mRNA level of *Vipr2* was observed before 4 weeks old and in females. In contrast, there were no differences in DNA methylation levels at the CpG islands of the *Vipr2* gene in the NAc between WKYs and SHRs.

**Conclusion:**

DNA methylation was not correlated with upregulation of *Vipr2* mRNA in SHRs. Further studies are needed to clarify the involvement of *Vipr2* mRNA upregulation in ADHD‐like symptoms.

## Abstract 2

Expression of the VIP/PACAP receptor gene, *Vipr2*, was selectively upregulated in the nucleus accumbens of spontaneous hypertensive rats (SHRs), an animal model of ADHD, with no changes observed in DNA methylation levels at the CpG islands of the gene. This region‐specific overexpression may contribute to ADHD‐related behaviors in SHRs.

## Graphical abstract

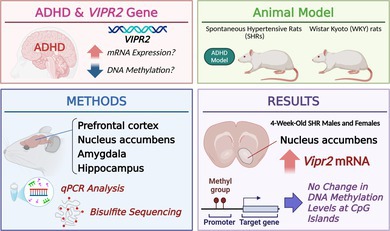

## 1 Introduction

Vasoactive intestinal peptide (VIP) receptor 2 (VPAC2), encoded by the *VIPR2* gene, is a seven‐transmembrane heterotrimeric G‐protein‐coupled receptor that binds two neuropeptides with high affinity: VIP and pituitary adenylate cyclase‐activating polypeptide (PACAP). VPAC2 is widely distributed throughout the body. In peripheral tissues, it is highly expressed in the heart, lungs, gastrointestinal tract, urogenital tract, pancreas, and other organs. There, VPAC2 exhibits vasodilatory, smooth muscle relaxant, and insulin secretion‐promoting effects [1, 2, 3, 4, 5, 6]. In the central nervous system, VPAC2 is primarily expressed in the thalamus, the suprachiasmatic nucleus, and the cerebral cortex. It has been reported to regulate circadian rhythms and control synaptic plasticity [7, 8]. Microduplications of the *VIPR2* gene are a known risk factor for schizophrenia [9, 10, 11, 12] and were detected more frequently in autism spectrum disorder compared with controls [9]. Single‐nucleotide polymorphism of *VIPR2* is also associated with mood disorders [13]. In animal studies, overactivation of VPAC2 during the early postnatal period decreases synaptic proteins in the prefrontal cortex (PFC) and causes prepulse inhibition deficit [14]. VPAC2‐deficient mice show impaired extinction of cued fear memory and abnormal dendritic morphology in the prelimbic and infralimbic cortices [15]. These reports have suggested that abnormalities in VPAC2 signaling, especially in early life, may be linked to various neurodevelopmental and psychiatric disorders.

Attention‐deficit/hyperactivity disorder (ADHD) is a developmental disorder characterized by inappropriate attention, impulsivity, and hyperactivity that affects approximately 3%–7% of school‐aged children [16]. With early‐onset behavioral and psychological symptoms, ADHD often persists into adulthood, and approximately 2%–5% of adults suffer from ADHD [17, 18, 19]. The precise etiology of ADHD is still not well understood. Although ADHD undoubtedly has high heritability, several researchers have reported an interaction between genetic and environmental factors [20, 21, 22] and have supported the epigenetic hypothesis of ADHD etiology [23, 24, 25, 26, 27]. Recently, blood DNA methylomes in adults who were malnourished during the first year of life revealed a weak positive correlation between DNA methylation in the *VIPR2* gene and Connors Adult ADHD Rating Scale scores [28]. A methylomic analysis of salivary DNA in children with ADHD showed DNA hypomethylation at two cytosine–phosphate–guanine (CpG) islands in the *VIPR2* gene [29]. Although the extent to which DNA methylation in peripheral tissue reflects DNA methylation in the brain is unknown, these methylome studies have implied that abnormal VPAC2 signaling, via alterations in the methylation and transcription of the *VIPR2* gene, may lead to the development of ADHD.

To date, many studies on the etiology of ADHD using animal models have been carried out. One of the most widely used animal models of ADHD is the spontaneous hypertensive rat (SHR), an inbred strain derived from the Wistar Kyoto rat (WKY). SHRs begin to exhibit hyperactivity [30, 31], and inattention or impulsivity [32, 33] starting from 3 to 4 weeks old, compared with WKYs. The drugs used clinically to treat ADHD, such as psychostimulants, ameliorate the hyperactivity of SHRs [34]. These reports suggest high face and predictive validity of the SHR as an ADHD model. However, there are no studies about the *Vipr2* gene in SHRs. In this exploratory study, we investigated whether there are any differences in the messenger RNA (mRNA) expression and DNA methylation levels of the *Vipr2* gene in the brain regions responsible for emotional and cognitive function, such as the PFC, nucleus accumbens (NAc), amygdala (Amy), and hippocampus [35, 36], between WKYs and SHRs using a relatively small sample size.

## 2 Materials and Methods

### 2.1 Animals

All animal experimental procedures used in this study were approved by the committee for Ethical Use of Experimental Animals at Setsunan University and were conducted in accordance with the ethical guidelines for the Journal of Pharmacological Sciences [37], *Guide for the Care and Use of Laboratory Animals* (National Research Council Institute for Laboratory Animal, 1996), and ARRIVE guidelines 2.0 [38]. Every effort was made to minimize animal suffering and limit the number of animals used. WKYs (WKY/NCrlCrlj) and SHRs (SHR/NCrlCrlj) were obtained from Charles River Laboratories Japan Inc. (Yokohama, Japan). Male and female 1–4‐week‐old WKYs and SHRs bred at the animal breeding facility of Setsunan University were used. The rats were housed in cages (26 × 38 × 20 cm^3^) with their mother and littermates before weaning (3 weeks old) or in groups of 3–5 animals after weaning. All rats were housed under a standard 12 h light/dark cycle (lights on at 8:00 a.m.) at a constant temperature of 23°C ± 1°C, with free access to food and water throughout the study. A total of 56 rats were used.

### 2.2 Total RNA Isolation and Reverse Transcription

The brain tissue was sectioned into 1 mm slices using Brain Matrices (ASI Instruments, Warren, MI, USA). Using the Rat Brain Atlas [39] as a reference, the PFC, NAc, and Amy were sampled with a 3 mm diameter Miltex Biopsy Punch (Integra LifeSciences, Princeton, NJ, USA), and the entire hippocampus was excised with a scalpel. Total RNA was isolated using TRIzol reagent (Invitrogen, Carlsbad, CA, USA) according to the manufacturer's protocol. Briefly, tissues were sonicated in 1 mL TRIzol reagent by a sonicator (#UR‐21P; Tomy Seiko, Tokyo, Japan), and the samples were incubated for 5 min at room temperature. Then, 0.2 mL chloroform was added, and the sample was shaken by hand for 15 min. After incubation for 3 min at room temperature and centrifugation at 12 000 × *g* for 15 min at 4°C, the aqueous phase of the sample was placed into a tube, and isopropanol was added to the aqueous phase. The mixture was incubated for 10 min at room temperature and centrifuged at 12 000 × *g* for 10 min at 4°C. Then, the supernatant was removed, and the pellet was washed with 75% ethanol. After centrifugation at 7500 × *g* for 5 min at 4°C, the pellet was air dried for 10 min and dissolved in 20 μL nuclease‐free water. Total RNA (1 μg) was used to perform reverse transcription with ReverTra Ace (Toyobo, Osaka, Japan) according to the manufacturer's protocol. Briefly, the 20 μL reactions were incubated for 40 min at 42°C and 5 min at 99°C, and then preserved at 4°C.

### 2.3 Quantitative Polymerase Chain Reaction (PCR)

Quantitative PCR was performed with Thunderbird qPCR Mix (Toyobo) and the primers indicated in Table 1, using a Thermal Cycler Dice Real Time System Single (Takara Bio, Shiga, Japan). The calibration curve is shown in Appendix S1. Changes in gene expression were calculated relative to the endogenous *Actb* (β‐actin) standard.

**TABLE 1:** List of primer sequences used for real‐time quantitative PCR analysis.

Gene	Forward primer sequence (5′–3′)	Reverse primer sequence (5′–3′)
*Vip*	TCCATTCTAAATGGGAAGAGGA	TCACTTGGTTGTTTTCCTTCAA
*Adcyap1*	ACAGCGTCTCCTGTTCACCT	CCTGTCGGCTGGGTAGTAAA
*Adcyap1r1*	TACCTGTCGGTGAAGGCTCT	ATGAAGTTGCGAGTGCAATG
*Vipr1*	CGCAGCACGAGTGTGAGTA	GGTGAGGTTGTCCCACATCT
*Vipr2*	GTGCTGACCTGCTACTGCTG	GCGCATTTTGTCTCCTCTTC
*Actb* (β‐Actin)	ACCCACACTGTGCCCATCTA	GCCACAGGATTCCATACCCA

### 2.4 Bisulfite Sequence Analysis

Bisulfite sequence analysis was performed as previously described [40]. Briefly, genomic DNA was extracted from the NAc with DNA extraction buffer [150 mM NaCl, 10 mM Tris–HCl (pH 8.0), 10 mM EDTA, 0.1% SDS, 100 μg/mL proteinase K] and isolated with phenol saturated with TE buffer (Nacalai Tesque Inc., Kyoto, Japan), phenol–chloroform–isoamyl alcohol (25:24:1, pH 7.9) (Nacalai Tesque), and ethanol. Bisulfite treatment of DNA was performed using a MethylEasy Xceed Rapid DNA Bisulphite Modification Kit (Takara Bio). The bisulfite‐treated DNA was amplified by PCR with the primers shown in Table 2. PCR products were electrophoresed and extracted using NucleoSpin Gel and PCR Clean‐up (Takara Bio). The extracted DNA was cloned into a pGEM‐T Easy vector (Promega, Madison, WI, USA). Sequences of 8–10 independent recombinant clones containing an insert of the correct sequence were determined using SP6 primer by a DNA sequencing service (Fasmac, Kanagawa, Japan).

**TABLE 2:** List of primer sequences used in bisulfite sequence analysis.

CpG island	Forward primer sequence (5′–3′)	Reverse primer sequence (5′–3′)
1 Chr6:143937883‐143938411	TGTTAGGGAGAGGTTTTGTAAAAAG	TATCCCCAACTCCAAAAACACTA
2 Chr6:143951239‐143951514	AGGGGATTGGAAAGAAAAGATTTTAGAA	TCTCTCTCTCTCTCTCTCTCTCTATATATA

### 2.5 Statistical Analysis

**FIGURE 1:**
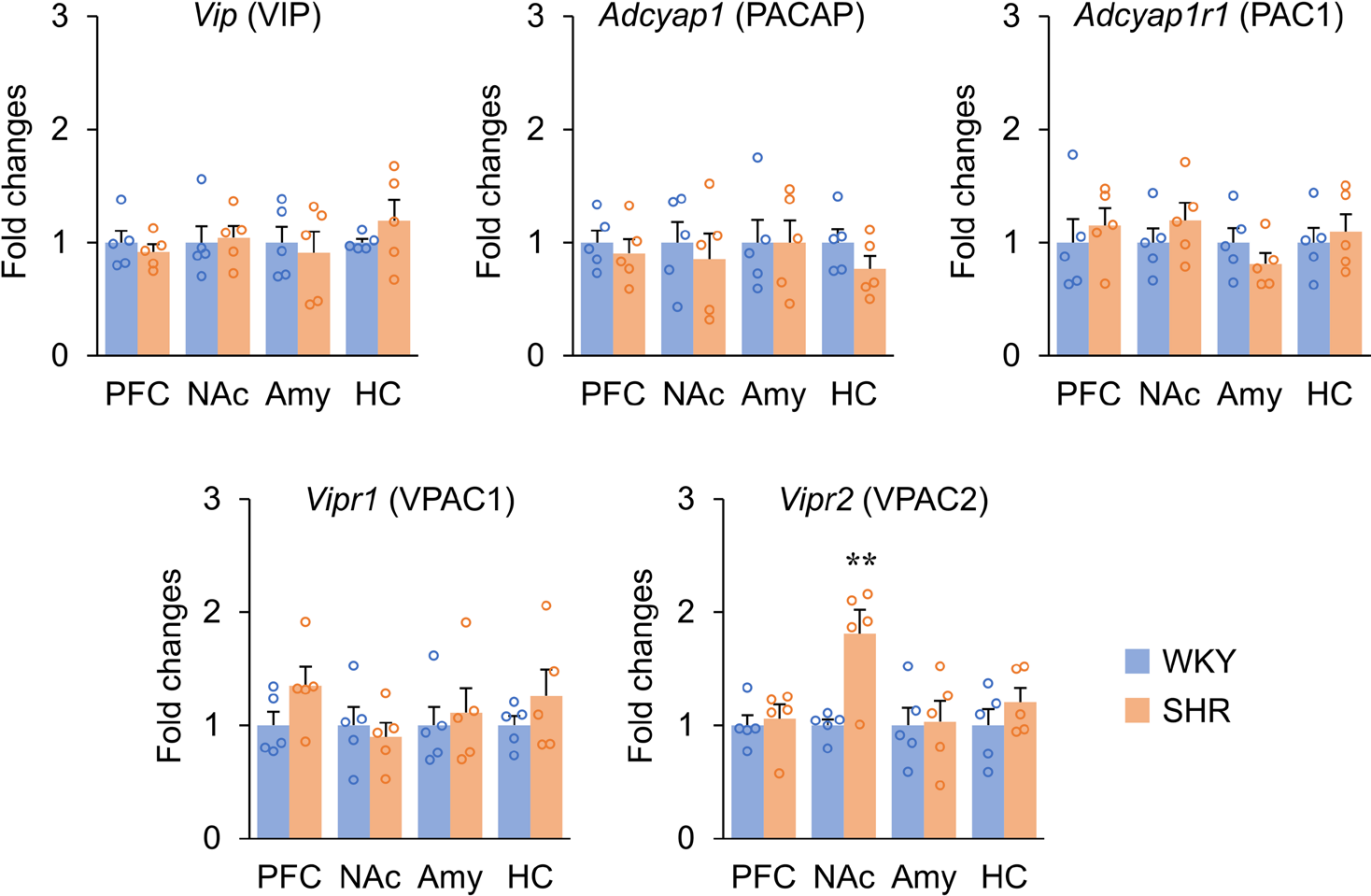
mRNA levels of vasoactive intestinal peptide (VIP)/pituitary adenylate cyclase‐activating polypeptide (PACAP) signal‐related genes in the prefrontal cortex (PFC), nucleus accumbens (NAc), amygdala (Amy), and hippocampus (HC) of 4‐week‐old male Wistar Kyoto rats (WKYs) and spontaneous hypertensive rats (SHRs). The mRNA levels of *Vip* (encodes VIP), *Adcyap1* (encodes PACAP), *Adcyap1r1* (encodes PAC1 receptor, one of the PACAP receptors), *Vipr1* [encodes VIP receptor 1 (VPAC1), one of the VIP/PACAP receptors], and *Vipr2* [encodes VIP receptor 2 (VPAC2), one of the VIP/PACAP receptors] were examined. Values are expressed as the mean + standard error of the mean of five rats. **p < 0.01 versus WKYs.

**FIGURE 2:**
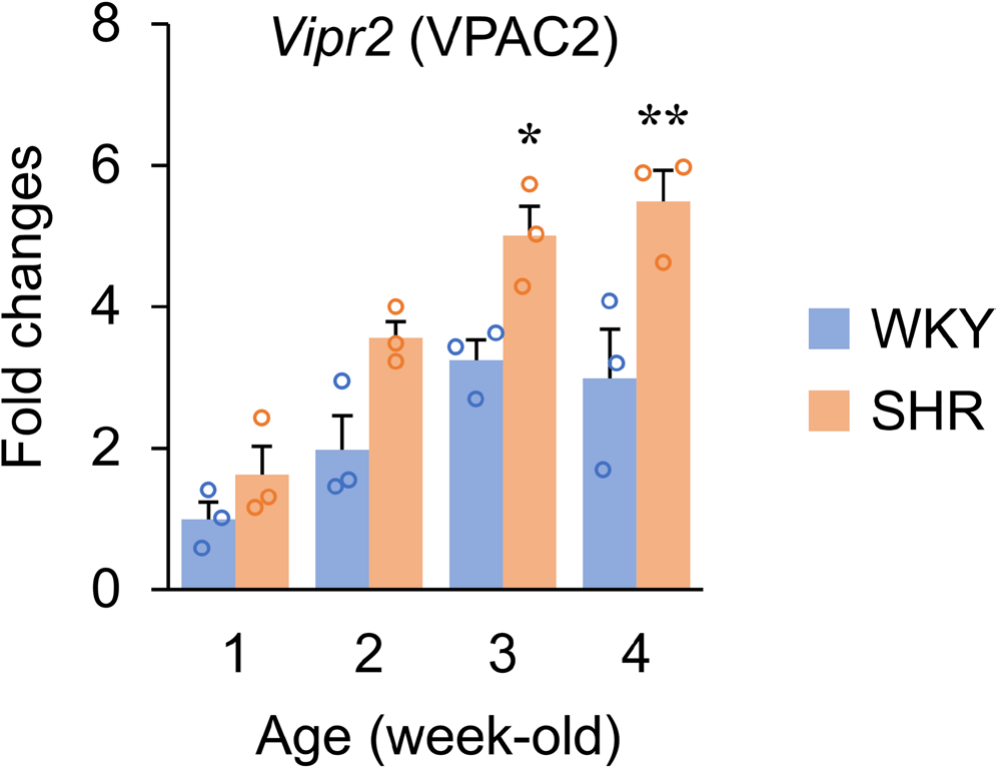
*Vipr2* mRNA levels in the nucleus accumbens in 1–4‐week‐old male rats. Values are expressed as the mean + standard error of the mean of three rats. *p_adj_ < 0.05, **p_adj_ < 0.01 versus Wistar Kyoto rats (WKYs). *p*_adj_ indicates *p*‐value adjusted by Bonferroni correction for multiple comparisons. SHR, spontaneous hypertensive rat.

**FIGURE 3:**
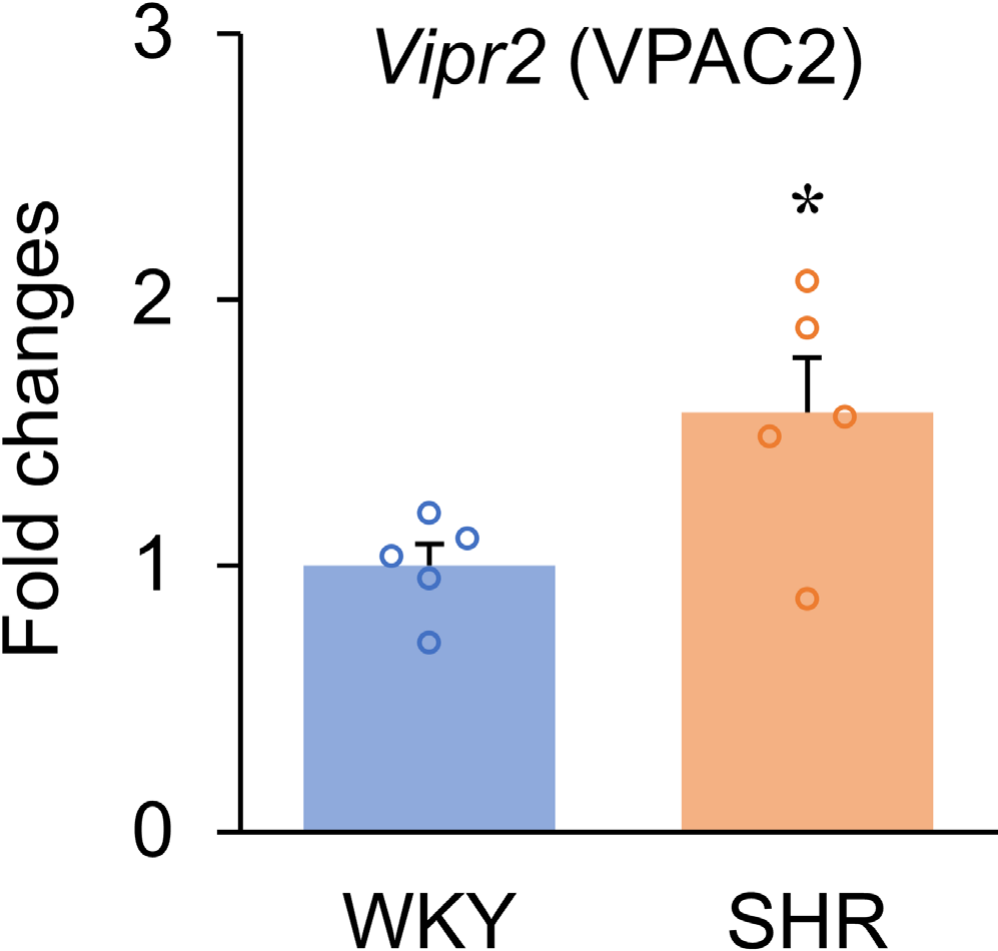
*Vipr2* mRNA level in the nucleus accumbens in 4‐week‐old female rats. Values are expressed as the mean + standard error of the mean of five rats. *p < 0.05 versus Wistar Kyoto rats (WKYs). SHR, spontaneous hypertensive rat.

**FIGURE 4:**
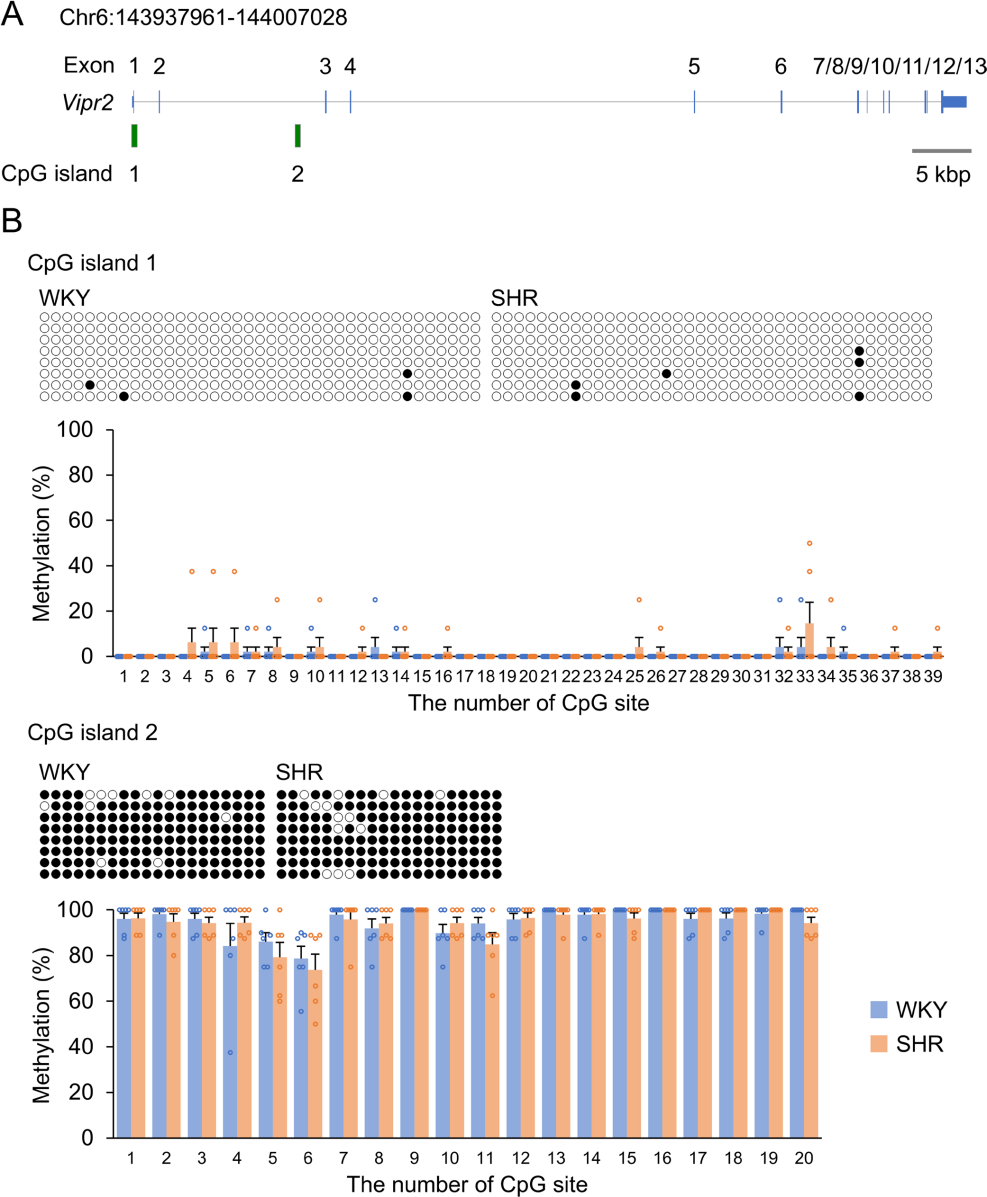
DNA methylation levels at the cytosine–phosphate–guanine (CpG) islands of the *Vipr2* gene in the nucleus accumbens in 4‐week‐old male rats. (A) Gene structure of the rat *Vipr2* gene. There are 13 exons and 2 CpG islands in the rat *Vipr2* gene. Exons for putative non‐coding genes and untranslated regions are shown as thin blocks, and exons for coding sequences as thick blocks. The horizontal bar indicates the length of 5000 base pairs. The image was adapted from the UCSC Genome Bioinformatics Site (http://genome.ucsc.edu/) [41]. (B) Representative DNA methylation patterns and DNA methylation levels at CpG islands 1 and 2 are shown. DNA isolated from the nucleus accumbens was bisulfite‐treated, and the CpG island 1 and 2 regions, containing 39 CpGs and 20 CpGs, respectively, were amplified by polymerase chain reaction. In each rat, 8–10 clones of these amplicons were sequenced, and the methylation levels in the CpG islands were recorded. The methylated CpG sites are indicated by black circles, and the nonmethylated CpG sites by white circles, for each clone. Values are expressed as the mean + standard error of the mean of six rats. SHR, spontaneous hypertensive rat; WKY, Wistar Kyoto rat.

All data are expressed as the mean + standard error of the mean. The data in Figures 1, 3, and 4 were analyzed using Student's *t*‐test. The data in Figure 2 were analyzed using two‐way analysis of variance (ANOVA), with strain and age as factors. Although the interaction between strain and age was not statistically significant, Bonferroni‐corrected simple main effects tests were performed to evaluate strain differences at each age, which were of primary biological interest. All analyses were performed using StatView 5.0J for Apple Macintosh (SAS Institute, Cary, NC, USA). A value of *p* < 0.05 was considered statistically significant.

## 3 Results

### 3.1 mRNA Levels of VIP/PACAP Signal‐Related Genes in the Brain

To analyze *Vipr2* mRNA levels comprehensively in brain regions involved in emotional control and the reward system, four major regions that constitute the emotional and cognitive‐behavioral network [35, 36] were selected: the PFC, which governs executive function; the NAc, which serves as the center for reward prediction and motivation; the Amy, which regulates emotional processing, such as fear and anxiety; and the hippocampus, which mediates learning and memory. *Vipr2* mRNA levels were higher in the NAc, but not in the PFC, Amy, or hippocampus, of SHRs compared with WKYs at 4 weeks old. There were no differences in *Vip* (encodes VIP), *Adcyap1* (encodes PACAP), *Adcyap1r1* (encodes PAC1 receptor, one of the PACAP receptors), or *Vipr1* (encodes VIP receptor 1 (VPAC1), one of the VIP/PACAP receptors) mRNA levels between WKYs and SHRs (Figure 1). Next, we assessed strain‐dependent differences in the NAc across ages (Figure 2). Although the sample size per group was relatively small (*n* = 3), the study was designed to primarily test main effects using a two‐way ANOVA. Significant main effects of strain (*F*_1,16_ = 21.10, *p* < 0.001) and age (*F*_3,16_ = 29.32, *p* < 0.001) were observed, with no significant strain × age interaction (*F*_3,16_ = 1.67, *p* = 0.21). SHRs had significantly higher *Vipr2* mRNA levels than WKYs at 3 and 4 weeks old (Figure 2). Similar to the results obtained for male SHRs, upregulated mRNA levels of *Vipr2* at 4 weeks old were also observed in female SHRs (Figure 3).

### 3.2 DNA Methylation Levels at CpG Islands in the *Vipr2* Gene

Two CpG islands (CG > 50%, observed CpG/expected CpG > 0.65) are located within the rat *Vipr2* gene (Figure 4A). Whereas CpG island 1 is located around the transcription start site, CpG island 2 is in the gene body. There are 39 and 20 CpG sites in CpG islands 1 and 2, respectively (Appendix S2: Figure S1). Bisulfite sequence analyses revealed no differences in the DNA methylation levels of CpG islands 1 and 2 in the NAc between male WKYs and male SHRs at 4 weeks old (Figure 4B).

## 4 Discussion

The SHR, an inbred genetic strain derived from the WKY, is widely used as an animal model of ADHD because SHRs show hyperactivity in an open‐field test [30, 31], and inattention or impulsivity [32, 33], compared with WKYs. These abnormal behaviors begin to appear at 3–4 weeks old [30, 42]. SHRs also develop hypertension at approximately 5–6 weeks old at the earliest [43]. In this study, to avoid any influence of hypertension, rats under 4 weeks old were used as an animal model of ADHD.

ADHD is thought to be linked to dysfunction in brain regions involved in reward, such as the NAc [44, 45]. A study using NAc slice cultures suggested that presynaptic regulation of dopamine release is altered in SHRs, causing downregulation of the dopamine system [46]. A mapping analysis of Fos protein, a neuronal activity marker, indicated that the number of Fos‐immunoreactive cells was greater in the core part of the NAc in SHR/Izm than in WKY/Izm animals at 5–6 weeks old, after the open‐field test [47]. These reports suggest differences in NAc function between WKYs and SHRs, and point to the significance of the NAc in the abnormal behaviors of SHRs. In this study, *Vipr2* mRNA levels were upregulated in the NAc of SHRs compared with WKYs (Figure 1). Upregulation of *Vipr2* mRNA levels was observed during the juvenile stage but not during the early postnatal period (Figure 2). In addition, there was no difference in the upregulation of *Vipr2* mRNA levels between the sexes (Figure 3), similarly to hyperlocomotion (Appendix S2: Figure S2). Abnormalities in VPAC2 signaling in early life may cause several neurodevelopmental and psychological abnormalities [9, 10, 11, 12, 13, 14, 15]. In particular, generating a human *VIPR2* duplication in mice results in increased dopamine D_2_ receptor immunoreactivity and dopamine contents in the dorsal striatum, as well as in cognitive, sensorimotor gating, and social behavioral deficits [48]. Genetic removal of VIPR2 transgene expression in dopamine D_1_ receptor‐expressing neurons rescued the dopamine D_2_ receptor abnormality and multiple behavioral deficits, suggesting a pathogenic role of VIPR2 overexpression in dopaminoceptive neurons. It is still unknown whether VPAC2 signaling is directly related to the accumbal dopamine system, but increased *Vipr2* mRNA expression may affect NAc function in SHRs.

DNA methylation is a mechanism potentially mediating the interaction between genetic and environmental factors. Therefore, to clarify the etiology of diseases on the basis of gene–environment interactions, it is worthwhile investigating DNA methylation patterns. Bisulfite sequencing revealed no differences in the methylation levels of the *Vipr2* gene between WKYs and SHRs (Figure 4B), unlike methylome analyses of individuals with ADHD. The human *VIPR2* gene has six CpG islands; one CpG island is located around the transcription site, and the others are in the gene body [41]. In humans, DNA hypomethylation has been observed at the two CpG islands in the salivary DNA of children with ADHD [29]. However, the sequences of those CpG islands are not highly conserved between the human *VIPR2* gene and the rat *Vipr2* gene. These findings suggest that the epigenetic alterations observed in the peripheral tissues of individuals with ADHD may not be directly recapitulated in the brain of SHRs.

This study has several limitations. First, the sample size is small due to the exploratory nature of the study, as well as ethical limitations and feasibility issues. Additionally, the littermate effect was not investigated. These factors have the potential to impact the reproducibility of the results. The current experimental conditions have limited statistical power and are susceptible to variability and outliers. Thus, there is uncertainty in reproducibility. Second, although we observed alterations in *Vipr2* mRNA levels, we did not examine protein expression. Therefore, it is unclear whether the VPAC2 function is altered in SHRs. Third, we did not examine any behaviors other than spontaneous locomotor activity in SHRs. Consequently, it is unclear which behaviors in SHRs are linked to increased *Vipr2* levels in the NAc. Since we did not analyze *Vipr2* mRNA and spontaneous motor activity in the same rats in this study, we could not perform a correlation analysis between VPAC2 expression and hyperlocomotion. We recently developed brain‐penetrating, VPAC2‐selective antagonist peptide KS‐133 nanoparticles [49]. To clarify the causal relationship between increased VPAC2 expression in the NAc and behavioral alterations in SHRs, it is necessary to examine the effects of VPAC2 antagonists on behaviors. Finally, our analysis of DNA methylation focused solely on whether it is altered in CpG islands within the *Vipr2* gene. Therefore, genome‐wide or region‐wide epigenetic alterations cannot be excluded. Additionally, since species‐specific differences in DNA methylation patterns are well‐documented, direct comparisons should be interpreted with caution.

In conclusion, we found that *Vipr2* mRNA levels were specifically increased in the NAc of SHRs, but *Vipr2* DNA methylation did not change. This exploratory, hypothesis‐generating study requires further validation with larger samples. Future studies are needed to determine how the increased VPAC2 expression impacts ADHD‐like symptoms.

## Author Contributions

**Ryota Araki:** conceptualization, investigation, visualization, data curation, funding acquisition, writing – original draft. **Ayami Kita:** visualization, data curation. **Yukio Ago:** conceptualization, supervision, methodology, funding acquisition, writing – review and editing. **Takeshi Yabe:** resources, supervision.

## Funding

This work was partially supported by grants from JSPS KAKENHI (grant numbers 20K07119, 23K06159 [R.A.], and 24K02185 [Y.A.]) and JSPS Program for Forming Japan's Peak Research Universities (J‐PEAKS) (grant number JPJS00420230011 [Y.A.]).

## Disclosure

Declaration of generative AI and AI‐assisted technologies in the writing process: The authors have nothing to report.

## Ethics Statement

All animal experimental procedures used in this study were approved by the committee for Ethical Use of Experimental Animals at Setsunan University and were conducted in accordance with the ethical guidelines for the Journal of Pharmacological Sciences, *Guide for the Care and Use of Laboratory Animals* (National Research Council Institute for Laboratory Animal, 1996), and ARRIVE guidelines 2.0.

## Consent

The authors have nothing to report.

## Conflicts of Interest

The authors declare no conflicts of interest.

## Supporting information


**Appendix S1:** npr270143‐sup‐0001‐AppendixS1.docx.

**Appendix S2:** npr270143‐sup‐0002‐AppendixS2.docx.

**Appendix S3:** npr270143‐sup‐0003‐AppendixS3.xlsx.

## Data Availability

All raw data and analyses in the manuscript are available in Appendix S3.
