# Supplementary material for: Vipr2 Gene Expression Is Upregulated in the Nucleus Accumbens of Spontaneous Hypertensive Rats During Early Life
Source: Neuropsychopharmacol Rep. 2026 Jun 17;46(2):e70143. doi: 10.1002/npr2.70143 (PMC13275169; doi:10.1002/npr2.70143)

**Supplementary information 1**

***Calibration curve in quantitative PCR***

| Legend | Target | R^2^ | Efficiency | Calibration Curve |
| --- | --- | --- | --- | --- |
| 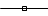 | *Vip* | 0.994 | 91.9 | Y = -3.532 * LOG(X) + 39.49 |


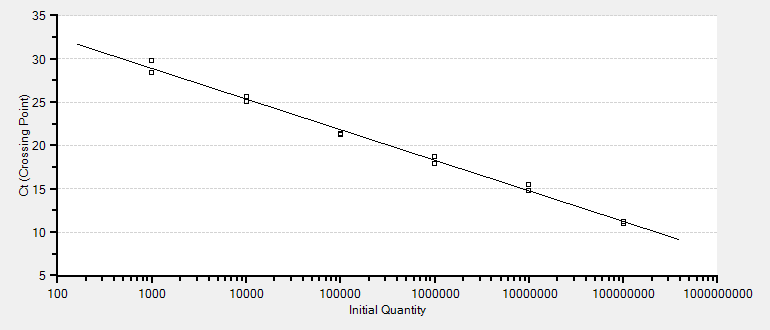


| Legend | Target | R^2^ | Efficiency | Calibration Curve |
| --- | --- | --- | --- | --- |
| 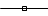 | *Adcyap1* | 0.991 | 99.9 | Y = -3.325 * LOG(X) + 40.17 |


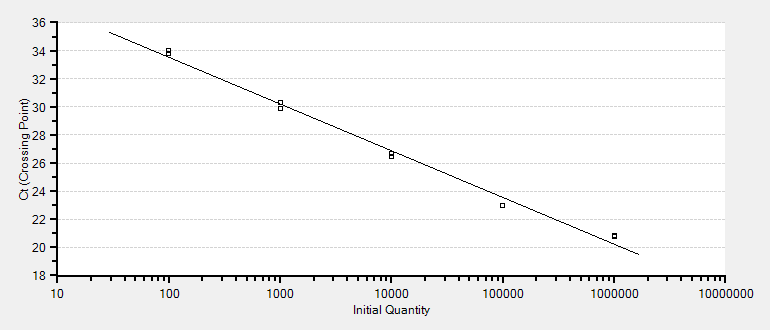


| Legend | Target | R^2^ | Efficiency | Calibration Curve |
| --- | --- | --- | --- | --- |
| 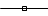 | *Adcyap1r1* | 0.988 | 97.7 | Y = -3.378 * LOG(X) + 29.45 |


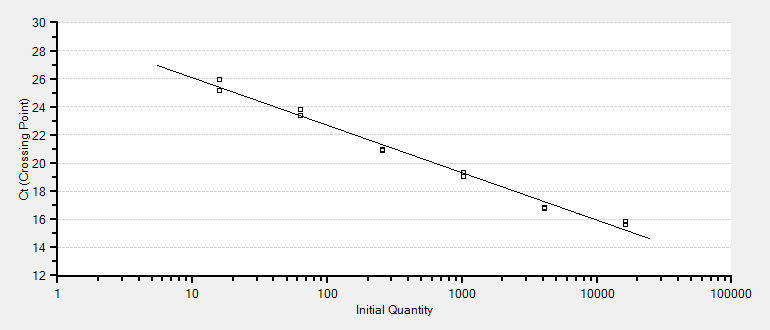


| Legend | Target | R^2^ | Efficiency | Calibration Curve |
| --- | --- | --- | --- | --- |
| 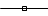 | *Vipr1* | 0.991 | 98.9 | Y = -3.349 * LOG(X) + 49.56 |


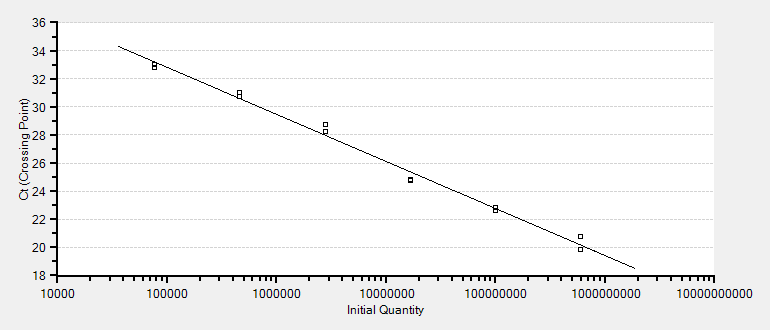


| Legend | Target | R^2^ | Efficiency | Calibration Curve |
| --- | --- | --- | --- | --- |
| 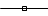 | *Vipr2* | 0.996 | 91.8 | Y = -3.536 * LOG(X) + 37.37 |


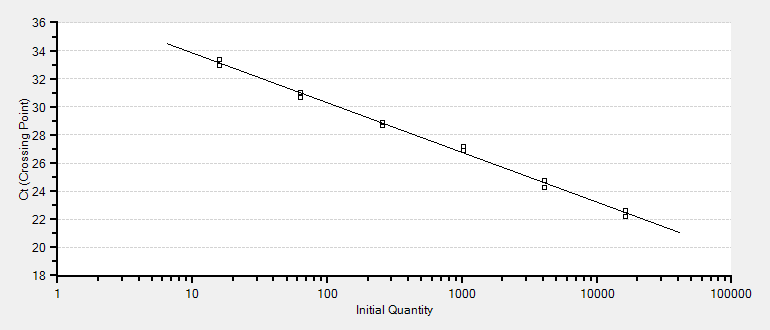


| Legend | Target | R^2^ | Efficiency | Calibration Curve |
| --- | --- | --- | --- | --- |
| 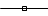 | *Actb* | 0.990 | 99.7 | Y = -3.329 * LOG(X) + 36.50 |


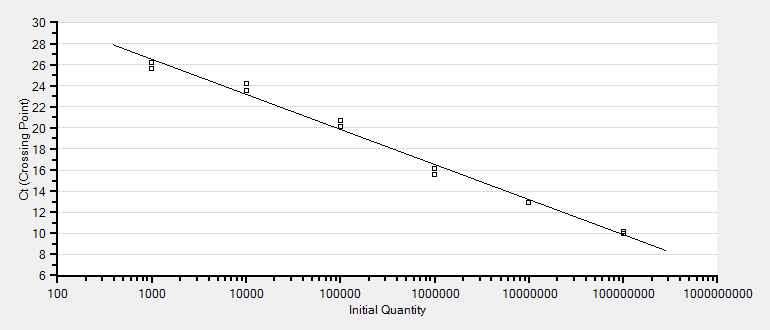

Supplement: Supplementary file 1 — Appendix S1: npr270143‐sup‐0001‐AppendixS1.docx. [file NPR2-46-e70143-s001.docx]
