# Supplementary material for: Vipr2 Gene Expression Is Upregulated in the Nucleus Accumbens of Spontaneous Hypertensive Rats During Early Life
Source: Neuropsychopharmacol Rep. 2026 Jun 17;46(2):e70143. doi: 10.1002/npr2.70143 (PMC13275169; doi:10.1002/npr2.70143)
Supplement: Supplementary file 2 — Appendix S2: npr270143‐sup‐0002‐AppendixS2.docx. [file NPR2-46-e70143-s002.docx]

**Supplementary information 2**

**Supplementary Methods**

*Locomotor activity in a novel field*

Locomotor activity was measured using ANY-maze video tracking software (Stoelting, Wood Dale, IL, USA). Each rat was placed individually in a novel clear Plexiglas cage (30 × 30 × 30 cm^3^), and the total distance traveled was analyzed for 60 min.

*Statistical analysis*

All data are expressed as the mean + standard error of the mean. The data in Fig. S2 were analyzed by Student’s *t*-test. A value of *P* < 0.05 was considered significant.

**Supplementary Figures**

**
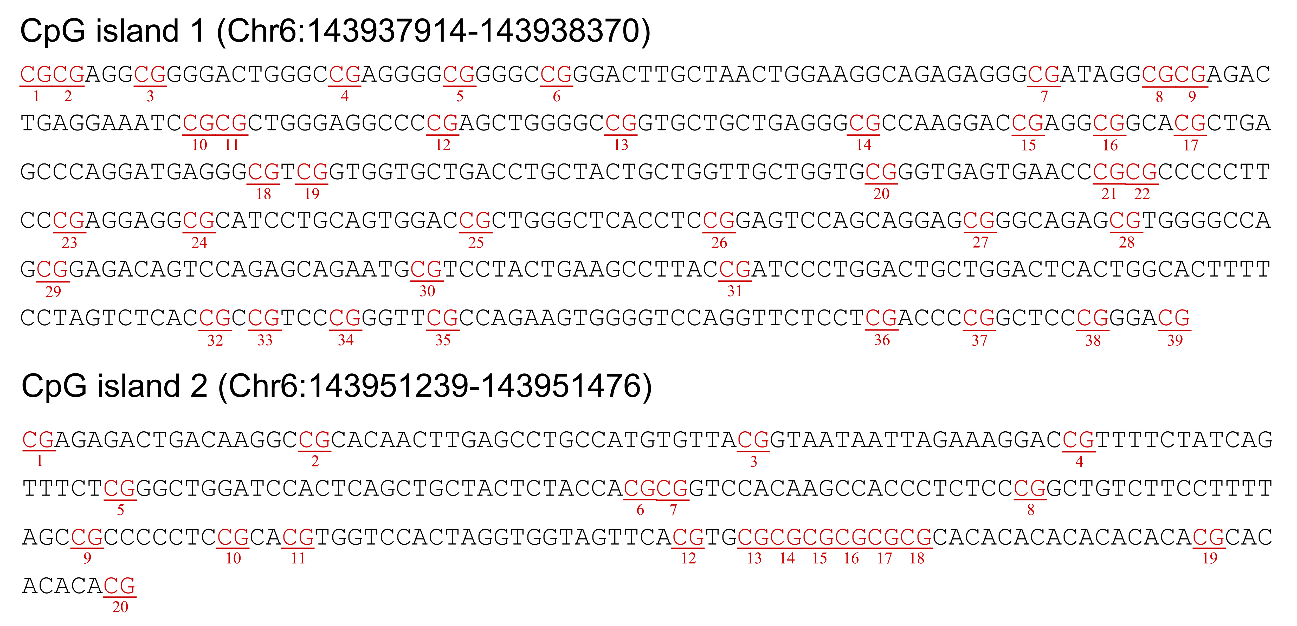
**

**Fig. S1.** DNA sequences of cytosine–phosphate–guanine (CpG) islands 1 and 2. The CpG sites in the CpG islands are underlined and numbered. There are 39 and 20 CpG sites in CpG islands 1 and 2, respectively.

**
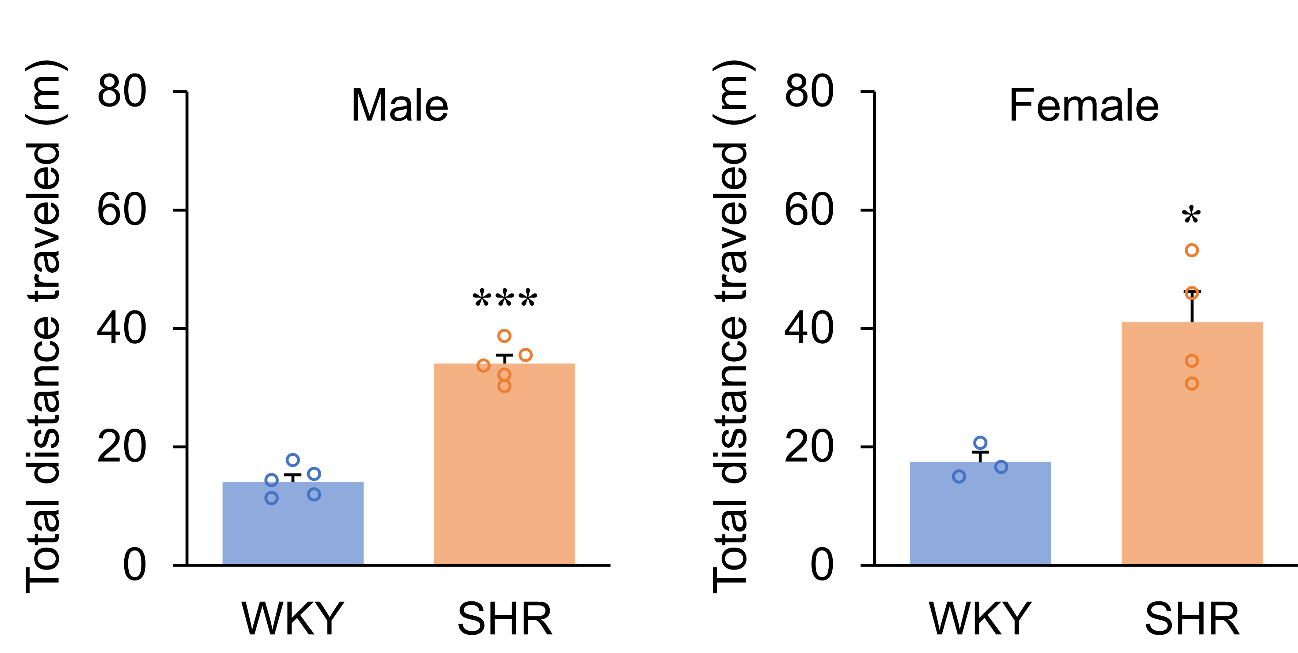
**

**Fig. S2.** Spontaneous locomotor activity of Wistar Kyoto rats (WKYs) and spontaneous hypertensive rats (SHRs) in a novel field. SHRs were more hyperactive than WKYs (both sexes). Values are expressed as the mean + standard error of the mean of 3–5 rats. ^*^*P* < 0.05, ^***^*P* < 0.001 vs. WKYs.
